# Optimizing hyperparameters of deep reinforcement learning for autonomous driving based on whale optimization algorithm

**DOI:** 10.1371/journal.pone.0252754

**Published:** 2021-06-10

**Authors:** Nesma M. Ashraf, Reham R. Mostafa, Rasha H. Sakr, M. Z. Rashad

**Affiliations:** 1 Computer Science Department, Faculty of Computers and Information Sciences, Mansoura University, Mansoura, Egypt; 2 Information Systems Department, Faculty of Computers and Information Sciences, Mansoura University, Mansoura, Egypt; Vellore Institute of Technology: VIT University, INDIA

## Abstract

Deep Reinforcement Learning (DRL) enables agents to make decisions based on a well-designed reward function that suites a particular environment without any prior knowledge related to a given environment. The adaptation of hyperparameters has a great impact on the overall learning process and the learning processing times. Hyperparameters should be accurately estimated while training DRL algorithms, which is one of the key challenges that we attempt to address. This paper employs a swarm-based optimization algorithm, namely the Whale Optimization Algorithm (WOA), for optimizing the hyperparameters of the Deep Deterministic Policy Gradient (DDPG) algorithm to achieve the optimum control strategy in an autonomous driving control problem. DDPG is capable of handling complex environments, which contain continuous spaces for actions. To evaluate the proposed algorithm, the Open Racing Car Simulator (TORCS), a realistic autonomous driving simulation environment, was chosen to its ease of design and implementation. Using TORCS, the DDPG agent with optimized hyperparameters was compared with a DDPG agent with reference hyperparameters. The experimental results showed that the DDPG’s hyperparameters optimization leads to maximizing the total rewards, along with testing episodes and maintaining a stable driving policy.

## 1. Introduction

Reinforcement Learning (RL) is a machine learning category, which should achieve the highest cumulative reward through interactions with an unknown environment. There is a trend to blend this category with deep learning, which led to significant advancements in different domains. RL has steadily improved because of the prominence of deep neural networks (DNN), leading to the emergence of Deep Reinforcement Learning (DRL), which has outperformed humans in various games in the last few years [1–4]. DRL has been broadly used for addressing multiple challenges in speech recognition [5], computer vision [6], natural language processing [7], and other AI models [8, 9].

DRL has been integrated with a Deep Q-Network (DQN) framework, where DNN offered the fundamental of Q-learning [1]. DQN demonstrated exceptional outputs in fifty variants of Atari games and contributed to developing various DRL systems [10–12]. DQN was concerned only with those tasks where there are minor and discrete states, as well as action spaces. Various RL tasks involve continuous states as well as action spaces. Though DQN might have accomplished continuous tasks by converting the continuous spaces into discrete ones, which in turn, will increase the unpredictability of the overall control mechanism. To address this issue, the Deterministic Policy Gradient (DPG) algorithm [13] coupled with DNN techniques, was deemed appropriate, resulting in the Deep Deterministic Policy Gradient (DDPG) algorithm [14]. However, DDPG is affected by inadequate exploration and unstable training occasionally [15].

A set of parameters must be predefined to ensure that the DDPG algorithm can explore and learn on its own during the interaction with a complex environment in a continuous control problem. These parameters, also known as hyperparameters, include neural network size, learning rates, exploration, and others. In the training phase, they are not automatically tuned, where developers would select them based on their experience. The outcome of the learning process and the interactions with the environment, as well as the required learning time, highly depends on the choice of the hyperparameters. Because of this, it is critical to accurately choose the best hyperparameters for enhancing the model’s performance. Each environment has its hyperparameters that suit its nature and complexity. An ordinary way of selecting those hyperparameters is to manually search for suitable ones, which demand expertise to find robust hyperparameter sets. However, it is not easy to find out the best hyperparameters [16]. An automatic hyperparameter search process offers has become essential as it enables a DRL algorithm to produce the optimal solution regarding any predefined problem. First, any application would be applied with a guarantee that a DRL algorithm will function at its best, as a user is not reliant on sophisticated personal experience regarding the tuning of hyperparameters. Second, the optimality-based problem solving using a DRL algorithm can be advanced into true automation, as only the identification of the optimal hyperparameters enables the DRL algorithms to deliver optimal results regarding the given task without any prior knowledge to the environment, and completely through trial and error.

Recently, optimization has become one of the most interesting topics in different life aspects and nature-inspired meta-heuristic optimization algorithms that are shown to be one of the promising optimization techniques. These algorithms have been utilized with AI methods because they: (i) are easy to implement and rely on rather simple concepts; (ii) do not require gradient information; (iii) can avoid local optima; and (iv) are utilized in multiple problems covering different research areas. Nature-inspired meta-heuristic algorithms resolve optimization problems by simulating physical or biological phenomena. These algorithms can be categorized into three main classes: swarm-based, evolution-based, and physics-based methods.

Evolution-based methods are motivated by the rules of the evolutionary process. Physics-based methods mimic the rules of physics evident in nature. Nature-inspired methods contain swarm-based methodologies that mimic the communal conduct of clusters of animals. Conventionally, swarm-based techniques have some better Properties than evolution-based algorithms. For instance, swarm-based algorithms save the data of search avenues over the next iterations. On the other hand, approaches based on evolution eliminate all details as soon as a new population is formed. Swarm-based algorithms generally contain reduced operations compared with evolutionary strategies (selection, crossover, mutation, elitism) and are comparatively easy to execute.

The most popular swarm-based optimization algorithms are Particle Swarm Optimization (PSO) [17] that is motivated by the societal conduct of bird flocking, Ant Colony Optimization [18], which is motivated by societal conduct of ants as they operate and perform in an ant colony. Recently, the Whale Optimization Algorithms (WOA) [19] which motivated by whales’ potential for chasing target prey. WOA can efficiently solve various issues and was extended with many updates from 2016 till today. The research for improving the WOA by extending the original work with new features or create a hybrid algorithm between WOA and other swarm-based optimization techniques is presented strongly in academic research.

WOA and its extensions are utilized in various domains, industrial applications, and academic theories. They have been confirmed with outstanding results in Electrical and Electronics Engineering [20–22], Economic Scheduling [23], Civil Engineering [24], Fuel and Energy [25, 26], Reduce the burden of the DNN features extraction from the dataset [27] and solving resource allocation problems in wireless networks [28].

In this paper, the DDPG algorithm [9] was adopted, which combines the ideas of DPG, actor-critic algorithms, and deep Q-learning. The DDPG agent was aimed to control an automated vehicle. Autonomous driving (AD) was selected as the field of interest in this paper because its state space is exceptionally complex, and action space is continuous. Besides that, a neat fine control is required. In addition, in a dynamic world, autonomous driving vehicles often have to ensure practical stability. These aspects make the AD task very challenging even for DRL algorithms that can tackle continuous spaces like DDPG. AD is an active research point in computer vision and control systems. In the industry, Google, Tesla, NVIDIA, Uber, and Baidu, are some of the companies devoted to developing advanced AD cars because they can benefit humans in the real life. The annual social benefits of AD systems, if widely adopted, are expected to hit approximately $800 billion by 2050, thanks to traffic reduction, reduced road fatalities, lower energy usage, and improved efficiency due to the reallocation of driving time [29]. In this context, respectful efforts have been put into the research to provide a reliable and safe experience to the future of AD for both connected vehicles [30, 31] and ego vehicles [32, 33]. On the other hand, the DRL technique can be seen as a promising technique to be applied in the field of AD.

To prevent physical damage, the Open Racing Car Simulator (TORCS) was chosen as the test environment in this paper. To learn the policy in TORCS, first, a set of appropriate sensor information was selected as inputs from TORCS. Based on these inputs, a rewarder inside TORCS has been adopted that was proven to encourage DDPG agents to maintain a promising driving policy in previous work.

Meanwhile, to make the perfect fit of DDPG in the TORCS environment, this paper aims to optimize a set of five critical hyperparameters of the DDPG algorithm using WOA. The optimized hyperparameters are (actor learning rate, critic learning rate, discount factor, learning rate of target networks, batch size). Those hyperparameters control the performance of the overall system. The WOA was chosen as the metaheuristic optimization technique in this paper due to its promising results. Because of the arbitrary structure of the optimization algorithm, achieving a suitable equivalence between exploitation and exploration in the improvement of any metaheuristic algorithm is a topmost task. Through its exploitation, exploration, and ability to get rid of local minima, WOA has the highest significance among the different optimization approaches [34]. Thanks to the whales’ location updating process, the WOA has a significant exploration capability. The whales are forced to travel randomly around each other during the first step of the algorithm. The algorithm then instructs the whales to update their positions frequently and travel along a spiral-shaped route in the direction of the best path found thus far. The WOA avoids local optima since these two stages are performed independently and in half iteration each. However, since most other optimization algorithms (such as PSO and GSA) do not have operators to consecrate a particular iteration to the exploration or the exploitation so the probability of falling into local optima is more likely increased [35].

To demonstrate the effectiveness of the proposed method, we evaluate our agent in training mode within TORCS. The results were compared with another DDPG agent’s performance, which uses a set of hyperparameters that was suggested by an expert. The results show that our agent had outperformed its competitor in terms of maximizing the accumulated rewards along with test episodes with a non-trivial difference.

This article makes the following contribution:

- Surveys the latest and most outstanding DRL research in the field of AD and state the research efforts which have been put into optimizing the DRL algorithm’s hyperparameters.
- targeting a set of five hyperparameters of the DDPG algorithms to be optimized which are known to be the most critical for the learning process efficiency [14].
- Applying WOA to find the optimal values of the selected hyperparameters to allow our agent to apply the continuous control within the TORCS environment.
- Comparing the performance of the DDPG agent that using the optimized hyperparameters with another DDPG agent which uses a set of hyperparameters proposed by an expert and shows that the proposed hyperparameters maximized the total rewards gained along with test episodes within the TORCS environment.

The remainder of this paper is organized as follows. In section 2, the background is given. The related work is discussed in section 3. The proposed approach is introduced in depth in section 4. Section 5 discusses the proposed method’s experimental findings. Section 6 concludes the paper.

## 2. Background

This section presents a brief background of Reinforcement Learning, the Deep Deterministic Policy Gradient algorithm (DDPG), Whale Optimization Algorithm (WOA), and The Open Racing Car Simulator (TORCS).

### 2.1 Reinforcement learning

RL [36] is a subdomain in machine learning, and it utilizes reward points explicitly obtained by the environment for learning a policy. Policy here is referring to the process of mapping observations (states) to actions. In the traditional perspective of reinforcement learning, an agent intends to learn an ideal policy given the environment E in discrete time steps. The Markov decision process (MDP) is formally used to describe RL problems. RL problem consists of the tuple (*S*,*A*,*P*,*R*,*γ*) where S refers to the state space, A is the action space, *P*(*s*_*t*+1_|*s*_*t*_, *a*_*t*_) is a transition function that predicts the next state (*s*_*t*+1_) given a current state-action pair (*s*_*t*_, *a*_*t*_), R is the reward function that defines the immediate reward r(s_t_, a_t_) achieved at each state-action pair, and finally, *γ* ϵ [0, 1] denotes a discount factor that gives more value to the reward given to the agent now more than the rewards that will be collected way in the future. Eq (1) describes the accumulated reward obtained by an agent starting from time step t along the trajectory of interactions with an environment:

$$R_{t}=\sum_{i=t}^{T}\gamma^{\left(i-t\right)}r(s_{i},a_{i})$$ (1)

When the agent optimizes the expected accumulated reward E [R_t_], the ideal strategy (policy) π^∗^ is expected to be reached.

### 2.2 Deep Deterministic Policy Gradient algorithm (DDPG)

DDPG [14] is an off-policy algorithm based on the DPG [13] method. As the name refers, the DDPG algorithm uses deep learning (represented here in DNN) to estimate the policy function μ deterministically besides approximating an action-value function *Q*(*s*, *a*).

The key features of the DDPG procedure are explained next. Initially, the DDPG algorithm uses the actor-critic framework [36]. It implies the presence of two segments, the actor as well as the critic. The actor preserves a policy. The policy gets a state in the form of input and produces an action as its output. The critic approximates the action-value function, which becomes beneficial for evaluating the fitness of the actor. Moreover, the algorithm uses two sets of DNN for the actor and the critic. There are main networks (main actor μ with weights θ and main critic Q with weights ω) and target networks (target actor $\overset{´}{\mu }$ with weights $\overset{´}{\theta }$ and target critic $\overset{´}{Q}$ with weights $\overset{´}{\omega }$). At last, the algorithm utilizes a stochastic gradient [13] for the updating of main network weights and a soft updating rule for the target network weights.

The DDPG algorithm eventually receives the replay buffer feature from (DQN) [1] to address computational resources’ consumption issues. Because DDPG is an off-policy algorithm, the replay buffer may be huge, enabling the algorithm to take advantage of training in randomly sampled mini-batches throughout unrelated environmental interactions, which maintains the concept of i.i.d (independent identically distributed) supposition. A deterministic policy makes the training more stable for continuous domains. The "soft" updating rule, which is utilized for updating the target networks, also increases the training process’s stability.

**Fig 1:**
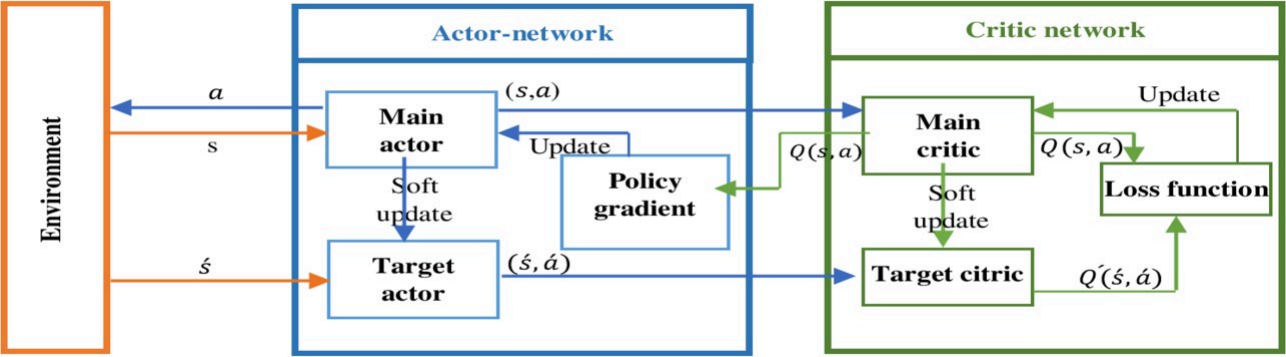
Diagram of deep deterministic policy gradient algorithm (DDPG).

This type of model is sufficiently adequate to demonstrate a tricky task as a continuous control problem. When the learning has been accomplished, the actor meets the ideal policy. (Fig 1) shows a Diagram of the DDPG algorithm.

Algorithm#1 DDPG algorithm

Input ← actor networks weights ***θ*** and critic networks weights ***ω***, replay buffer **R**, Max episodes **E**_**max**_, Max steps **T**

Output ← optimal policy π*

1. Begin

2.     Randomly initialize main critic network ***Q***(***s***, ***a***) and main actor-network **μ(s)** with weights ***ω*** and ***θ***

3.     Initialize target critic network $\overset{´}{Q}(s,a)$ and target actor-network $\overset{´}{\mu }(s)$ with weights $\overset{´}{\omega }$ and $\overset{´}{\theta }$

4.     Initialize replay buffer R

5.     For ***i* = 1 *to* E**_**max**_ do

6.       Initialize action exploration noise process Ɲ

7.       Receive initial state ***s***_**1**_ from the environment.

8.       For ***i* = 1** to ***T*** do

9.         Execute action ***a***_***t***_= μ $(s_{t}|\theta )+Ɲ$

10.         Observe reward ***r***_***t***_ and successor state ***s***_***t*+1**_

11.         Store experience (***s***_***t***_**, *a***_***t***_**, *r***_***t***_**, *s***_***t*+1**_) in R

12.         Sample random minibatch of N transitions from **R**

13.         Set $y_{i}=r_{i}+\gamma \overset{´}{Q}(s_{i+1},\overset{´}{\mu }(s_{i+1}|\overset{´}{\theta })|\overset{´}{\theta })$

14.         Update the critic by minimizing the loss L where $L=\frac{1}{N}\sum_{i}{\left(y_{i}-Q(s_{i},a_{i}|\omega )\right)}^{2}$

15.         Update the actor using the sampled policy gradient:

$\nabla_{\theta }\mu {|}_{s_{i}}\approx \frac{1}{N}\sum_{i}\nabla_{a}Q(s,a|\omega ){|}_{s=s_{t},a=\mu (s_{t})}\nabla_{\theta }\mu (s|\theta ){|}_{s_{t}}$

16.         Update the target network weight according to the soft update rule

17.       End for

18.     End for

19. End

### 2.3 Whale Optimization Algorithm (WOA)

The original version of the WOA was demonstrated in [19] by Mirjalili and Lewis. The effectiveness of the WOA was demonstrated in solving various issues [25, 37, 38]. The procedure is motivated by the potential of a whale to chase the target prey. The primary objective in WOA is to find out the target (optimal solution) by chasing it using the hunting strategy of the humpback whales. The hunting process of the humpback whales involved by first, surrounding the target, subsequently produces a net of bubbles to confine the target. Similar to other swarm-based optimization techniques, WOA depends on two strategies, which are exploration (seeking for the prey) and exploitation (hunting the prey) [19].

This combination of searching techniques for indicating the optimal solution ensures that the algorithm’s output is close to optimal solution among all possible solutions since exploration circumvents the local optima by expanding the outcomes’ plurality [39]. The bubble-net system in humpback whales can be mathematically modeled as the following.

#### Step 1: Bubble net attacking method (exploitation phase)

There is two approaches for mathematically model the humpback whale’s bubble net behavior. The two approaches are described as follows:

1 **Encircling Prey**. When whales identify the target’s location, they create a circle to produce a net. Initially, the ideal solution’s position remains unknown; therefore, the WOA presupposes the current leading candidate as the current best solution. Next, other searching agents are utilized to alter their positions to arrive at the current best agent’s location. The strategy is manifested, as mentioned in Eqs (2) and (3) below:
$$\vec{X}(t+1)=\vec{X^{*}}(t)-\vec{A}.\vec{D}$$ (2)
$$\vec{D}=|\vec{C}.\vec{X^{*}}(t)-\vec{X}(t)|$$ (3)
  In the equations, $\vec{X^{*}}(t)$ denotes the whale’s previous best location at step t. $\vec{X}(t+1)$ the current position of a whale, $\vec{D}$ is the distance from the whale point to the target, and the || indicates the absolute value. ‘A,’ as well as ‘C,’ serve as coefficients, and their computations are given below:
$$\vec{A}=2.\vec{a}.\vec{r}-\vec{a}$$ (4)
$$\vec{C}=2.\vec{r}$$ (5)
  In Eq (4), *a* parameter is proportionately reduced in the range of 2 to 0 in all phases (exploration and exploitation); thereby, $\vec{A}$ is a harmonic range that decreases by $\vec{a}$. $\vec{r}$ denotes a random vector in [0,1]. The updated location of all search agents can be approximated between their initial locations and the best agent’s location.

Algorithm#2 Whale Optimization Algorithm

**Input** ← number of whales (**N)**, the maximum number of iterations (***T***_**max**_**)**

**Output** ← best search agent X*

1. Begin

2.    Set number of whales.

3.    Initialize whales’ positions randomly.

4.    Calculate the fitness of each whale.

5.    Find the best whale X*

6.    While (t < ***T***_**max**_)

7.        For (***i* = 1 *to N***) do

8.            Update parameters (***a*,*A*,*c*,*l*** and ***p***)

9.            If (***p***<**0.5**)

10.                If (|A| <1) then Update current whale position by Eq (2)

11.                Else if (|A| >= 1) then Update current whale position by Eq (8)

12.                End if

13.            Else if (p >= 0.5)

14.                Update current whale position by Eq (6)

15.            End if

16.          End for

17.        Force whales’ position to remain inside the search boundaries

18.        Calculate the fitness of each whale

19.        Update the current best whale X*

20.        *t* = *t*+1

21.    End while

22. End

2 **Spiral Updating Position.** The area between the whale positioned at (*X*,*Y*) and the target positioned at (X*Y*) is represented, then a helix-shaped movement is implemented by using the spiral equation as shown in Eq (6).
$$\vec{X}(t+1)=e^{bl}.\cos(2\pi l).\vec{D^{*}}+\vec{X^{*}}(t)$$ (6)
  where $\vec{D^{*}}=|\vec{X^{*}}(t)-\vec{X}(t)|$, b denotes the constant used to determine a logarithmic spiral shape and l denotes a random number in the range [−1, 1].
  When updating whale’s positions, there is a likelihood of 50 percent to choose among shrinking encircling mechanism and the spiral model as follows:
$$\vec{X}(t+1)=\left\{\begin{array}{l} \vec{X^{*}}-\vec{A}.\vec{D,}\;if\;p<0.5,\\ e^{bl}.\cos(2\pi l).\vec{D^{*}}+\vec{X^{*}}(t),\;if\;p\geq 0.5 \end{array}\right\}$$ (7)
  In the equation, p represents a random quantity within (0, 1).

#### Step 2: Search for prey (exploration)

During this stage, the whales essentially utilize a random search to explore the target. Hence, to force the agents to distance from the best whale, the ’A’ vector is supported with a random value not equal to 1. In this phase of exploration, the agents’ position is rearranged based on randomly choosing the search agent instead of looking for the best agent. The exploration strategy is beneficial for WOA addressing the issue of local optima. The exploration position update is demonstrated in Eqs (8) and (9).


$$\vec{X}(t+1)=\vec{X_{rand}}-\vec{A}.\vec{D}$$ (8)

$$\vec{D}=|\vec{c}.\vec{X_{rand}}-\vec{X}|$$ (9)

Where $\vec{X_{rand}}$ represents a random location (randomly selected whale) based on the current population.

### 2.4 The Open Racing Car Simulator (TORCS)

**Fig 2:**
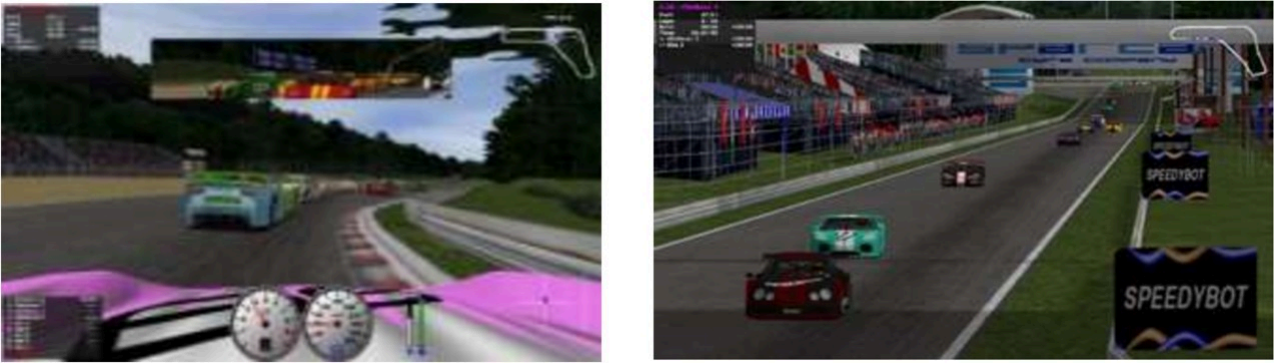
An example screenshot of TORCS.

The environment used for this paper is TORCS [40]. TORCS is a publicly available 3D racing game based on OpenGL technologies. TORCS has been widely used in academic research and has been chosen in this paper because it provides a realistic driving simulation. The simulator concept was first introduced in 1997 by Eric Espi and Christophe Guionneau. The simulator was coded in C++, and AI driving agents were developed using it over the years. TORCS executes detailed-oriented physics processes that consider various dimensions of real-life car driving, for instance, wheel movement, aerodynamics, car damages, and so forth. Therefore, TORCS enabled the development of highly trained agents. (Fig 2) shows an example screenshot representing TORCS.

In TORCS, the car driver receives a sense of the racing scene provided by numerous sensors to know the ego vehicle’s most recent position. Next, the controller executes the driving tasks, including brakes application, using the accelerator, and using the steering by adhering to a specific control policy. The driver uses a UDP connection to communicate with the race server. At each stage, the server transmits the latest updated environmental state and evaluates any response from the controller within 10 ms. In the case of no response, the previous response is considered. It is essential to state why TORCS is a very popular testbed for DRL:

- It provides good interactivity between the client (car controller) and the race server.
- In TORCS, it is possible to visualize neural networks’ learning over the training and assess the overall process. In contrast to other methods where one can only view the final results, this feature is very beneficial.
- It is also possible to visualize the stage when the neural network is stuck in local minima.

## 3. Related work

In this section, the recent research efforts in the domain of DRL for AD as well as optimization of DRL hyperparameters are presented.

### 3.1 DRL for autonomous driving

This section describes how different DRL algorithms dealt with the continuous control problem in AD. To simplify the process and enable DRL methodologies, which are effective in discrete action spaces exclusively (such as DQN), to function on such a domain as AD, an action space may be discretized uniformly as in [41]. Also, a Deep Q-Learning with filtered experience (DQFE) procedure was proposed with an extended experience replay strategy in [42]. The model has productively been trained on a routing technique with (TORCS).

However, Discretization has its drawbacks; it may result in a jerky or unstable driving policy. Another option to overcome this limitation is to use non-discrete values for actions directly by DRL methodologies. In this case, an agent’s training is accomplished explicitly through DRL that could handle the complex and continuous nature of the AD problem (such as DDPG). In this area, authors in [43] demonstrated the use of the DDPG algorithm to handle a real-size automated car.

Also, a temporal abstractions options framework was used to reduce the complexity concerned with the choice of actions for AD in [44], where agents choose options rather than direct low-level actions. The options constitute a subset of a policy as an extension of a basic action over several stages. Authors in [45] also adopt the (DDPG) algorithm to align DDPG strategies with TORCS and develop their reward function (which has been adopted in this paper). They also designed their network architecture for actors as well as critic within the DDPG framework. They present the network’s efficiency by showing both quantitative and qualitative results.

Authors in [46] utilized DQN for the refinement stage within Inverse-RL to obtain the rewards. This effort focused on learning humanistic lane change attempts. Authors in [47] indicated an expert demonstration from human drivers to optimize a solid driving policy. The method used was Maximum Entropy Inverse RL (IRL) so that the optimization of human-like and comfortable driving trajectories could be learned.

Model-based deep RL strategies are also suggested for training modes and methodologies in AD directly from raw pixel inputs [48–50]. For a detailed review of the Adoption of DRL in AD tasks, we refer the reader to [51, 52].

The objective of constructing a reward function for the DRL agents in AD is open to debate. Different criteria are integral components of a successful reward function design for DRL agents in AD tasks. These criteria are: distance traveled, car speed, maintaining the standstill state of the car, collision with scene objects and road walkers, infractions on the sidewalk, keeping in the lane, keeping smoothness and stability, and finally following traffic guidelines. In Table 1, a list of the research efforts that have been put into designing the reward function according to the criteria mentioned above is shown.

**Table 1:** Research efforts in designing reward function for the AD problem with different criteria.

REFERENCE	Aspects considered in designing reward function for AD							
	Traveled distance towards a destination	Car speed	Maintaining the standstill state of the car	Collision with scene objects and road walkers	Infractions on sidewalk	keeping in lane	Maintaining smoothness and stability	Following the traffic rules
[36]						✓		
[43]	✓				✓	✓		
[44]		✓				✓	✓	
[45]			✓				✓	
[46]				✓			✓	✓

From the literature survey, it has been observed that most of the works concentrate on applying DRL algorithms to AD by discretizing the action space uniformly which led to jerky driving policy, or by applying vanilla DDPG algorithm, which led to an acceptable performance that could be farther improved. Hence, to address this issue a hyperparameters optimization for the DDPG algorithm is performed in this paper to provide a stable driving policy for AD.

### 3.2 Optimization of DRL hyperparameters

This section deals with related studies concerned with hyperparameter’s optimization of different DRL methodologies.

Hyperparameter adaptation in DRL based on the Bayesian method was proposed in [53]. Where Chen et al. conducted the most thorough RL hyperparameters study, opting to use Bayesian optimization to configure the AlphaGo algorithm. Bayesian optimization facilitates an automatic solution to tune the game-playing hyperparameters of AlphaGo. The traditional methods can never achieve these results. Bayesian optimization improved the winning chances of AlphaGo and helped gain key data that can prove beneficial for constructing updated variants of self-play agents with Monte Carlo Tree Search (MCTS). However, this methodology necessitates conducting significant trials and requires advanced information. Moreover, these strategies can mostly adapt to one particular hyperparameter, and they are unable to adapt to a range of hyperparameters.

In [54] Liessner et al. describe a model-based hyperparameter optimization for DDPG that proved efficient in the industrial application settings. The optimization is extended with strictness on the available training time for the DDPG algorithm in the selected domain in their work. The authors showed that DDPG hyperparameters were optimized under hard time constrain. DDPG hyperparameters were also optimized in [55], where Authors used a Genetic Algorithm (GA) to identify adequate HER+DDPG hyperparameters. The used GA found the hyperparameters that requiring fewer epochs to learn better task performance. They utilized this approach on reach, fetch, push, slide, place, pick, and open processes in robotics manipulation jobs.

Authors in [56] concurrently proposed a method similar to population-based training (PBT). They indicated an alternate approach that is focused on the evolutionary mechanism, which is the OMPAC method. OMPAC was the first approach for executing DRL’s multiple hyperparameters adaptation by the population-based strategy. Authors in [57] also utilized the population-based neural network training (PBT) approach that essentially uses a static computational budget to optimize a population of models and their hyperparameters configuration to reach the best output. The proposed approach produced good outcomes in Machine Translation, DRL, and GANs. Nevertheless, PBT utilizes primitive stochastic perturbations for obtaining hyperparameter adaptation, which has been considered ineffective to track changes of potential temporary ideal hyperparameter configuration.

Recently, authors in [58] demonstrated a process of online hyperparameter adaptation for DRL. The efficient online hyperparameter adaptation method was an improved procedure of Population-based Training (PBT). An operation inspired by the GA called recombination operation is presented into the population optimization to accelerate the population’s convergence towards the best hyperparameter configuration. The authors confirmed this approach’s effectiveness and provided improved results compared to PBT, which is a classical approach consistent with their work.

Also, authors in [59] introduced the pioneering, confirmed, and effective Population-Based Training (PBT-style) procedure, named Population-Based Bandits (PB2). The procedure enables the identification of outstanding hyperparameter setups with fewer agents compared to PBT. Authors demonstrate with several RL trials, that PB2 can reach outstanding levels in a modest computational budget.

Strategies such as Population Based Training that are trained to learn optimal schedules for hyperparameters instead of fixed settings will produce promising outcomes, however, they are affected by sample ineffectiveness.

To address the limitation mentioned above, S. Paul et al. propose Hyperparameter Optimization on the Fly (HOOF) as a method to learn the hyperparameters of a policy gradient algorithm in [60]. A gradient-free procedure that needs only a single training execution to automate the hyperparameter adaptation that affects the policy gradient update. Their method was producing sample efficient and computationally effective algorithms that can be implemented easily. The findings within all used fields and algorithms indicate that the utilization of HOOF for learning the hyperparameter schedules results in quicker training with exceptional results.

An alternative approach was introduced in [61] to setting the TD’s λ hyperparameter, which is responsible for maintaining timescale concerning the TD updates. They reinforce the λ choice as a bias-variance trade-off in which the outcome is λ hyperparameter that results in the minimum Mean Squared Value Error (MSVE). Leave-One-Trajectory-Out Cross-Validation (LOTO-CV) was used for searching the domain of λ values. The approach highlighted that LOTO-CV could be executed effectively to fine-tune λ hyperparameter in an automated manner. However, these adaptation types often necessitate comprehensive practical skills and require significant computation power in various real-life scenarios.

From the literature survey, it has been observed that most of the works concentrate on applying grid search, Bayesian methods, or genetic algorithms to optimize different DRL hyperparameters, which shows some success but still has obvious drawbacks. Hence to achieve unprecedented results, a swarm-based metaheuristic optimization algorithm, which is the WOA, is proposed in this work to optimize the DDPG algorithm’s hyperparameters in the AD field.

## 4. Proposed framework

This section presents this paper’s primary contribution: where the WOA searches the space of DDPG hyperparameters trying to reach the hyperparameter set that maximizes the total rewards gained within the TORCS environment.

Following hyperparameters is the target set:

- actor learning rate *α*_*actor*_
- critic leering rate *α*_*critic*_
- discount factor ϒ
- *τ* parameter (the parameter used to soft updates both actor and critic target networks)
- Batch size

The selected range of all the hyperparameters is shown in Table 2.

**Table 2:** List of hyperparameters to be optimized with ranges.

parameter	range
*α*_*actor*_	[1e-04] – [1e-05]
*α*_*critic*_	[(1e-03) – (1e-4)]
discount factor ϒ	[0–1]
*τ* parameter	[(0) -(1e-02)]
Batch size	[32-512]

### 4.1 DDPG network structure

The training is done using two sets of networks, which are critic networks and actor networks. The actor networks input is a 29-dimensional state vector (selected from 18 different types of sensor inputs available in the TORCS environment), and the output is a 3-dimensional action vector. The output consists of 3 continuous actions, which are **Steering**, **Acceleration, and Brake**

Steering is a single unit with tanh activation function (in such, -1 means a max right turn and +1 means a max left turn). A single unit with a sigmoid activation function assigned for each of acceleration and brake separately (where 0 means no gas in the case of acceleration and no brake at all in the case of brake, 1 means full gas in the case of acceleration, and bull brake in the case of brake). Meanwhile, the critic network’s input is the state vector and the action vector together, and the output of the critic network is an action-value function *Q*(*s*, *a*). Only in the second hidden layer does the 3-dimensional action vector enter the critic network.

The actor and critic network configurations are shown in Table 3. There are two hidden layers of both actor and critic networks with 300 units and 600 units, respectively. Using the ADAM algorithm [62], the main network parameters are optimized. Meanwhile, the main network’s parameters are initialized at random.

**Table 3:** Actor and critic networks configuration.

name	actor	critic
Input layer	29 feature state vector	State vector and action vector (s_t_, a_t_)
1st fully-connected layer	300 Neurons	600 Neurons
2nd fully-connected layer	300 Neurons	600 Neurons
Output layer	3-dimensional action vector (at)	Q-value
Initial parameters	Uniformly random between [−3*e*^−3^, 3*e*^−3^]	Uniformly random between [−3*e*^−3^, 3*e*^−3^]
Optimizer	ADAM	ADAM

### 4.2 Training of DDPG networks

#### Critic network training

Critic networks comprise the main network and target network. A batch of samples is used for updating the main network at each discrete time stage. The minibatch sample acquires data from the replay memory. The batch size is determined based on the 5th value of each whale’s position vector (according to the arrangement of the hyperparameters to be optimized as whales’ positions). Particularly, the main critic network weights are optimized to minimize the loss as shown in Eq (10).


$$L=\frac{1}{N}\sum_{i}{\left(y_{i}-Q(s_{i},a_{i}|\omega )\right)}^{2}$$ (10)

Where L represent the loss and i indicates the ith sample in the batch and

$$y_{i}=r_{i}+\gamma Q^{\prime }(s_{i+1},\mu^{\prime }(s_{i+1}|\overset{´}{\theta })|\overset{´}{\omega })$$ (11)

where *γ* is the discount factor hyperparameter (gamma), Which defines how many time steps from the future the agent considers when picking the current action (this value strongly depends on the environment).

Target critic network weights are coupled with main critic network weights since it is updated using the soft-updating technique with learning rate *τ* as shown in Eq (12).


$$\overset{´}{\omega }\leftarrow \tau \omega +(1-\tau )\overset{´}{\omega }$$ (12)

#### Actor-network training

Similarly, the main actor network is updated by using the DPG theorem as shown in Eq (13).


$$\nabla_{\theta }\mu {|}_{s_{i}}\approx \frac{1}{N}\sum_{i}\nabla_{a}Q(s,a|\omega ){|}_{s=s_{i},a=\mu (s_{i})}\times \nabla_{\theta }\mu (s|\theta ){|}_{s_{i}}$$ (13)

Where ∇_*a*_*Q*(*s*, *a*|*ω*) is the gradient of the critic network parameters w.r.t. action *a* and ∇_*θ*_*μ*(*s*|*θ*) is the gradient of actor-network parameters w.r.t. actor parameter *θ*. The target actor is updated the same as the target critic using the soft-updating technique with the same learning rate *τ* as shown in Eq (14).


$$\overset{´}{\theta }\leftarrow \tau \theta +(1-\tau )\overset{´}{\theta }.$$ (14)

### 4.3 Learning process

**Fig 3:**
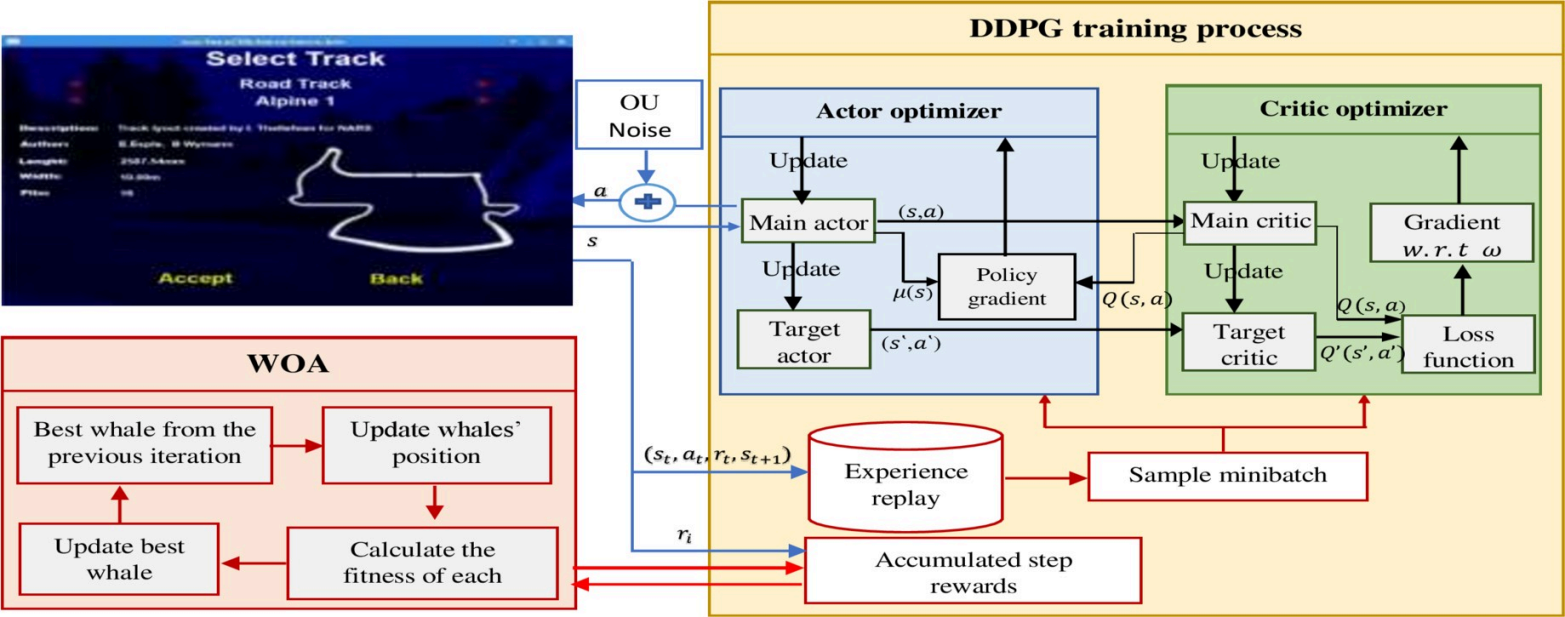
The workflow of the proposed DDPG+ WOA is applied to the TORCS environment.

The position vector of each whale is a combination of DDPG hyperparameters that needs to be optimized, arranged as follows: (*α*_*actor*_, *α*_*critic*_, *γ*, *batch size*, *τ*). Each whale is considered a DDPG agent, where each whale’s training process is done with the set of hyperparameters found in the corresponding whale’s position vector. Each whale’s fitness function is calculated via accumulating the total reward gained during the whale’s complete training process. Algorithm 3 explains the integration of DDPG-WOA for hyperparameter optimization and the whole hyperparameters optimization process is summarized in (Fig 3).

The training process of each whale described as follows:

1. At each step, the state of the environment (s_t_) is sent to the agent, and the actor returns the continuous values of (steering, acceleration, and break) as the action (a_t_) where *a*_*t*_ = *μ*(*s*_*t*_|*θ*)+*N*_*t*_ is the added noise for exploring the space of action using the Ornstein-Uhlenbeck method [63]).
2. TORCS transmit to the next state (s_t+1_) and return reward (r_t_) to the agent.
3. For further usage, the sampled information (s_t_, a_t_, r_t_, s_t+1_) is preserved in an experience replay buffer.
4. To train the networks, a randomly selected minibatch of size N from experience replay is used, as seen in section (4.2).
5. The step reward is accumulating along with the complete training episodes and used as each whale’s fitness function that must be maximized. At each iteration, the best whale X* with the best fitness is updating if a better whale were found, as shown in section (2.3).

Algorithm#3 WOA for optimizing DDPG hyperparameters

Input ← number of whales (**N)**, maximum iterations (***T***_**max**_), Max episodes (**E**_**max**_**),** Max steps **(T)**

Output ← optimized set of (***α***_***actor***_**, *α***_***critic***_**, *γ*, *batch size*, *τ***) hyperparameters.

1. Begin

2.    set number of whales **(N)**

3.    Initialize whales’ positions (***α***_***actor***_**, *α***_***critic***_**, *γ*, *batch size*, *τ***) randomly

4.    while (t < ***T***_**max**_)

5.      For (*i* = 1 *to* ***N***)

6.        Randomly initialize main critic network ***Q***(***s*, *a***) and main actor-network **μ(s)** with weights ***ω*** and ***θ***

7.        Initialize target critic network $\overset{´}{Q}(s,a)$ and target actor-network $\overset{´}{\mu }(s)$ with weights $\overset{´}{\omega }$ and $\overset{´}{\theta }$

8.        Initialize replay buffer R

9.        Set hyperparameters (***α***_***actor***_**, *α***_***critic***_**, *γ*, *batch size*, *τ***) as in position vector of the current whale

10.            For (***i* = 1 *to* E**_**max**_**) do**

11.                Initialize action exploration process Ɲ

12.                Receive initial state ***s***_**1**_ from environment

13.                For ***j*** =**1** to ***T*** do

14.                    Execute action ***a***_***t***_ = μ $(s_{t}|\theta )+Ɲ$

15.                    Observe reward ***r***_***t***_ a and successor state ***s***_***t*+1**_

16.                    Store experience (***s***_***t***_, ***a***_***t***_, ***r***_***t***_, ***s***_***t*+1**_) in R

17.                    accumulated step reward

18.                    Sample random minibatch of N transitions from R

19.                    Set $y_{i}=r_{i}+\gamma \overset{´}{Q}(s_{i+1},\overset{´}{\mu }(s_{i+1}|\overset{´}{\theta })|\overset{´}{\theta })$

20.                    Update the critic by minimizing the loss in Eq (10)

                    Update the actor using the sampled policy gradient as shown in Eq (13)

21.                    Update the target network weight according to Eq (12) and Eq (14)

22.                End for

23.            End for

24.            Set the fitness of each whale to the accumulated train rewards value

25.            Find the best whale X* with the highest fitness

26.            Update parameters (***a*,*A*,*c*,*l*** and ***p***)

27.            Update whales’ positions using WOA algorithm (algorithm#3)

28.        End for

29.        Update the current best whale X*

30.        t = t+1

31.    End while

32.    Return X*

33. End

## 5. Experiments

In this section the experimental analysis is presented, where the results of the optimized hyperparameters are shown and compared with the DDPG hyperparameters presented by Lillicrap in [14].

### 5.1 Experimental setting

All experiments were made on an Ubuntu 16.04 machine, with 8 cores CPU, 64 GB memory, and 4 GTX-780 GPU (8 GB Graphic memory in total). The DDPG replay buffer size is 100000 state-action pairs.

### 5.2 Training analysis

TORCS engine contains different modes. Generally, they can be divided into two kinds: training mode and compete mode. In compete mode, computer-controlled AI can be added into the game to race with our agent. Of course, other competitors’ existence will affect our car’s sensors and therefore affect the input state. For this reason, we select train mode for training where there are no other competitors in the race, and the view-angle is first-person. The selected training map is the Aalborg map. The WOA uses 8 whales over 10 iterations (all whales in each iteration run in parallel along with the eight cores). Each whale trains the DDPG algorithm on TORCS for 2000 episodes. If the car rushes out of the track or if the car is oriented in the opposite direction, the current episode will end. Therefore, the episode’s length is highly variated, a perfect model can play infinitely through one episode. Thus, it is essential to set a maximum length of an episode. Max steps count was set to 100000 steps per episode.

#### Reward (r)

TORCS does not have internal rewards; for this reason, a designed reward function must be provided. The reward should encourage the speed to increase along the track axis simultaneously the reward function must punish the speed vertical to the track axis at the same time.

We adopted the reward function design in [45]. Authors in [45] designed their reward function as shown in Eq (15).


$$R_{t}=V\times \cos\theta -V\times \sin\theta -V\times |trackpos|$$ (15)

Where *V*×cos*θ* denotes the speed that should be encouraged along the track. *V*×sin*θ* denotes the Vertical speed (the transverse velocity). The distance between the car and the track line is determined by |trackPos|. Both the second and third terms penalize the agent when the agent deviates from the middle of the lane.

#### State (s)

In TORCS, there are 18 different types of sensor input [64]. The used input sensors are shown in Table 4.

**Table 4:** The 29-dimensional state vector used in this paper.

Name	Range	Details
Angle	[-*π*,+*π*]	The angle between the position of the vehicle and the direction of the track axis
Track	(0,200)	19 range finder sensor vectors: each sensor returns the distance between the edge of the track and the car within a 200-meter range.
trackPos	(−∞,+∞)	Distance between a car and the axis of the track
speedX	(−∞,+∞)	Car speed along the car’s longitudinal axis (km/hour)
speedY	(−∞,+∞)	Car speed along the car’s transverse axis (km/Hour)
speedZ	(−∞,+∞)	Car speed along the car’s Z axis (km/Hour)
Wheel spin value	(0,+∞)	Vector of 4 sensors representing the rotation speed of wheels (rad/second)
Rotation per min	(0,+∞)	Number of rotation per minute of the car engine

#### Training performance of the best whale (X*)

Each optimization fills the DDPG algorithm’s memory (replay buffer) with sample episodes in the initial stage of execution. The learning process has not started yet in this phase. In the second step, the learning process continues relying on the sets of experiences in memory.

**Fig 4:**
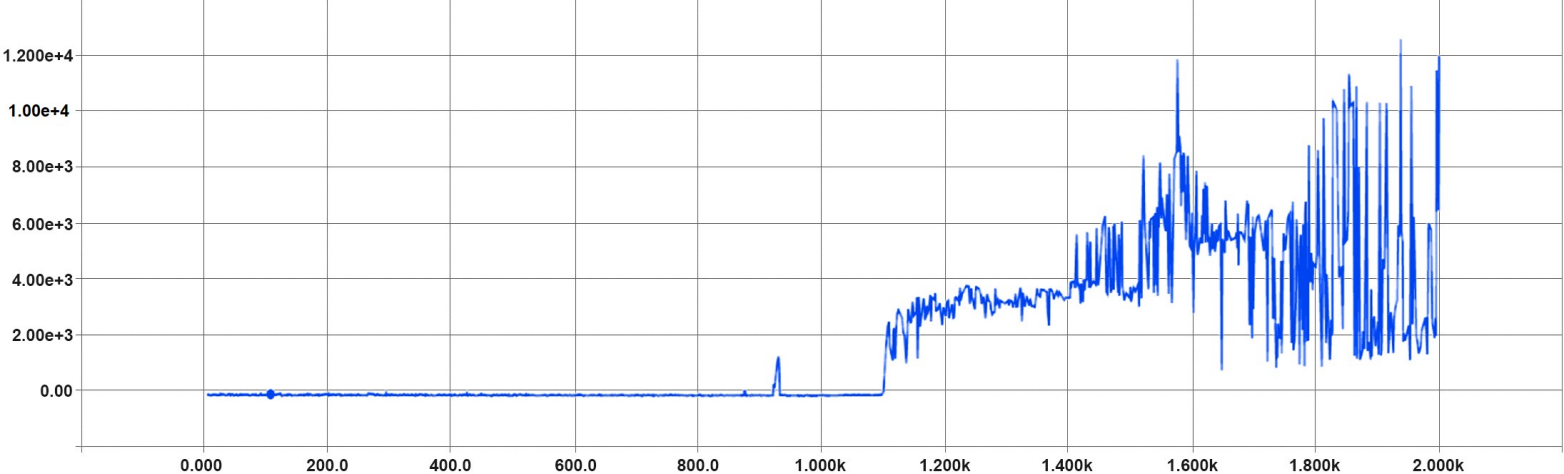
Optimized hyperparameters train total rewards per episode (x-axis shows the train episodes count and the y-axis shows the gained total rewards per episode.

**Fig 5:**
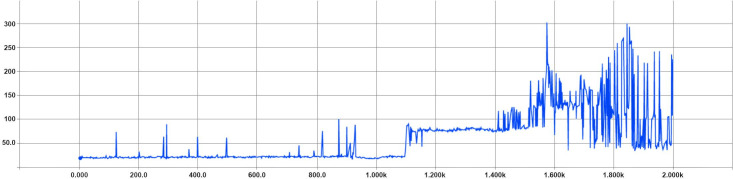
Optimized hyperparameters train total steps per episode (x-axis shows the episodes count and y-axis shows the total steps the agent spent in each episode).

We can see in (Fig 4) that the cumulative reward per episode is increasing as training progresses and also see in (Fig 5) that the overall steps per episode are increasing as well. This improvement is because the algorithm has improved and is less likely to fail or run out of track.

### 5.3 Validation map

**Fig 6:**
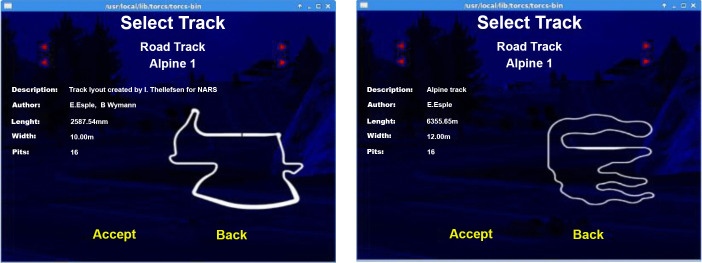
Train and evaluation maps on TORCS. (a) TRAIN Map Aalborg. (b) TEST Map Alpine 1.

The selected validation race map is (**Alpine 1**). Testing the agent in different tracks than the training track is a must to avoid overfitting where the AI simply memorizes the track. That is why Alpine 1 is used since it is three times longer than the training map and has lots of sharp turns. The train and validation track are shown in (Fig 6).

### 5.4 Evaluation of the hyperparameter optimization results

At this point, the performance of the optimized hyperparameters is analyzed in comparison to the expert hyperparameters. As a reference, the hyperparameters from the initial DDPG paper [14] are described. The reference hyperparameter set is not arbitrary selections, but they have already been optimized by the author (Lillicrap) and provide a good learned policy in different domains. Therefore, the optimized hyperparameter must exceed the already good selection of parameters.

**Fig 7:**
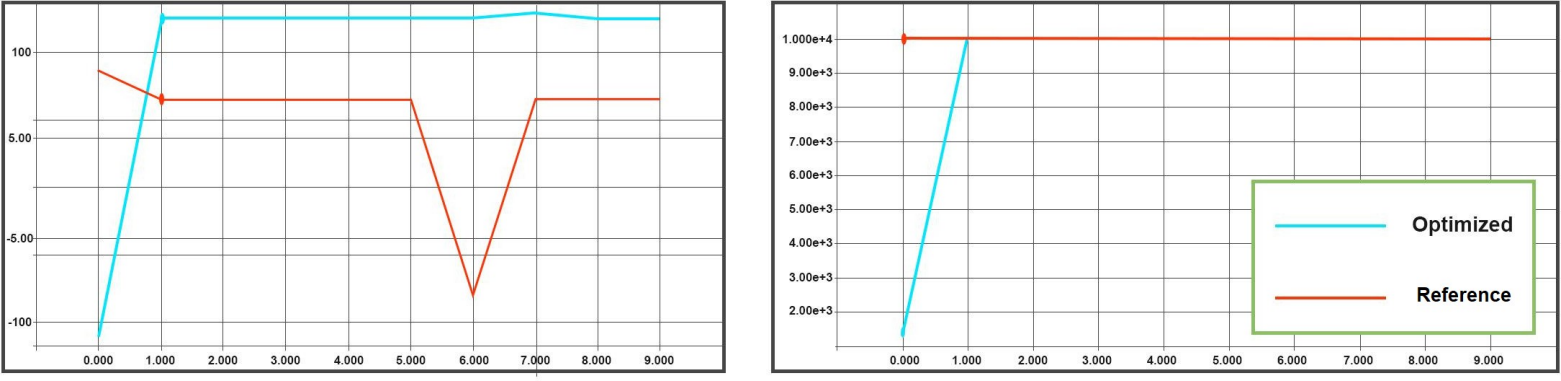
Optimal hyperparameters over ten runs, vs. reference hyperparameters. a) Optimal hyperparameters vs. reference total reward per episode (X-axis represent the episode count and Y-axis represent the accumulated reward), b) Optimal hyperparameters vs. reference total steps per episode (X-axis represent the episode count and Y-axis represent steps achevied).

For this purpose, testing both hyperparameters set (optimized and reference) is done on the validation track for ten episodes with max-steps equals 10000 steps per episode with the same termination conditions as in training. The results of the validation run deliver the values for (Fig 7). As stated before, the goal of the analysis in this paper is to finish the validation race on TORCS within the maximum total rewards comparing to the reference set of hyperparameters plus spend more steps or the same number of steps on the track. Since the reward function is designed to maximize longitudinal velocity, minimize transverse velocity, and also penalize the AI if it frequently drives the very off-center of the track, it is guaranteed that maximizing the number of steps spent on the track while maximizing the total rewards will result in a solid driving policy.

Table 5 lists the hyperparameters chosen by Lillicrap and the optimized values of hyperparameters.

**Table 5:** Original vs. optimal values of parameters.

hyperparameter	Reference value	Optimized value
*α*_*actor*_	1e-04	7e-04
*α*_*critic*_	1e-03	4.6e-03
ϒ	.99	.96
*τ*	.001	6.7e-03
Batch size	64	50

In Table 6, the max and min value of total travel distance, total rewards, and mean gain of the car controlled by the DDPG agent trained with reference hyperparameters and the DDPG agent trained with optimized hyperparameters among all 10 test runs are shown. The apparent improvement in total rewards gained by the optimized hyperparameters show that the model avoids the drunk driver attitude when the agent drives the car in an 8-shape [45].

**Table 6:** Original vs. optimal values of parameters test results among 10 test runs.

Hyperparameter set	Max mean gain among 10 runs	Min mean gain among 10 runs	Max travel distance among 10 runs (m)	Min travel distance among 10 runs (m)	Max total rewards among 10 runs	Min total rewards among 10 runs
reference	0.0053	-0.0039	10000 (m)	10000(m)	53.48	-170
Optimized	0.0385	-0.129	10000(m)	1310(m)	385	-39

### 5.5 Comparison

There are different works proposed in the literature that tried to optimize different RL algorithms hyperparameters [Table 7]. For example, Sehgal et al. [55] used a genetic algorithm (GA) to find the values of parameters used in (DDPG) combined with Hindsight Experience Replay (HER) to help speed up the learning agent. They used this method on fetch reach, slide, push, pick and place, and door opening in robotic manipulation tasks. Since the GA is probabilistic, they show results from 10 runs of the GA, and the results show that the optimized parameters found by the GA can lead to better performance. The learning agent can run faster and can reach the maximum success rate faster. The results show changes when only two parameters (out of the five target parameters) are being optimized. The results from optimizing all five parameters justify that GA found parameters outperformed the original parameters of the DDPG, indicating that the learning agent was able to learn faster. They state that the initial results bore the assumption that GAs are a good fit for such parameter optimization and the six manipulation tasks. They did not state the limitation of their method.

**Table 7:** Comparisons with recent state-of-art techniques.

*Technique*	*Optimization algorithm*	*DRL algorithm to be optimized*	*Target set of hyperparameters to be optimized*	*Environment*	*Results*
Sehgal et al. [55]	genetic algorithm (GA)	(DDPG) combined with Hindsight Experience Replay (HER)	discount factor; polyak-averaging coefficient; critic learning rate; actor learning rate; percent of times a random action is taken; standard deviation of Gaussian noise η	Robotic manipulation tasks.	Optimized parameters found by the GA can lead to better performance. The learning agent can run faster and can reach the maximum success rate faster.
Jaderberg et al. [57]	Population Based Training (PBT)	UNREAL on, Feudal Networks, and simple A3C agent	learning rate, entropy cost, and unroll length	DeepMind Lab levels, Atari games, StarCraft II	PBT results in the automatic discovery of hyperparameter schedules and model selection. It has the potential to reduce the computational resources for training.
Elfwing et al. [56]	Online Meta-learning by Parallel Algorithm Competition (OMPAC) method	Sarsa (λ)	learning rate α, discount factor γ, and λ (lambda parameter)	Stochastic SZ-Tetris, standard Tetris and Atari 2600.	OMPAC adaptation of the meta-parameters can significantly improve the learning performance when using suitable starting values of the meta-parameters
Proposed	WOA	DDPG	Actor learning rate, critic learning rate, discount factor, target networks learning rate, batch size.	The open racing car simulator (TORCS)	WOA can find hyperparameters that achieve the optimal strategy by maximizing the gained accumulated reward and training episodes. At the same time, maintain the same training steps compared against the DDPG agent with original hyperparameters.

Jaderberg et al. [57] present a population-based training (PBT). This simple asynchronous optimization algorithm effectively utilizes a fixed computational budget to jointly optimize a population of models and their hyperparameters to maximize performance. They demonstrate the effectiveness of PBT on DRL problems, showing faster wall-clock convergence and higher final performance of agents by optimizing over a suite of hyperparameters. In all cases, PBT results in the automatic discovery of hyperparameter schedules and model selection which results in stable training and better final performance. In all three test cases (DeepMind Lab levels, Atari games, StarCraft II), they show faster learning and higher performance across a suite of tasks, with PBT allowing discovery of new state-of-the-art performance and behavior, as well as the potential to reduce the computational resources for training. Results show that PBT increases the final performance of the agents when trained for the same number of steps, compared to the very strong baseline of performing a random search. Using PBT increases the final performance of UNREAL from 93% to 106% human performance. Since PBT is copying the weights of good performing agents during the exploitation phase, agents lucky in environment exploration are quickly propagated to more workers. StarCraft II showed how PBT improved A3C baselines from 36% human performance to 39% human performance when averaged over six levels.

S. Elfwing et al. [56] proposed the Online Meta-learning by Parallel Algorithm Competition (OMPAC) method. The idea behind OMPAC is simple. They aim to run several instances of an RL algorithm in parallel, with slight differences in the initial values of the meta-parameters. The OMPAC method is similar to the evolutionary process without the crossover operator but with two main differences from standard applications of artificial evolution. First, the goal is not to find the parameters that represent the optimal solutions directly; instead, the goal is to find the meta-parameters that enable RL agents to learn more efficiently. Second, the goal is not to find the best set of fixed parameters; instead, the goal is to adopt the values of the meta-parameters according to the current learning progress. The experiments in the two Tetris and the Atari 2600 domains showed that OMPAC adaptation of the meta-parameters could significantly improve the learning performance when using suitable starting values of the meta-parameters.

In case of our study, the WOA was selected as an appropriate swarm-based optimization algorithm since WOA has a high significance compared to other optimization approaches due to its exploitation, exploration, and ability to get rid of local minima [34]. WOA was selected to optimize 5 of the DDPG hyperparameters (actor learning rate – critic leering rate – batch size - discount factor – soft update parameter). Those hyperparameters must be predefined before training began and has the most critical role in the learning process. Our findings show that the WOA can find hyperparameters that achieve the optimal strategy by maximizing the gained accumulated reward and training steps as we investigated the applicability of the proposed method within an AD environment represented in the TORCS environment.

## 6. Conclusion

In this paper, we have presented the usability of WOA for optimizing reinforcement learning model hyperparameters. We evaluated its performance in optimizing the hyperparameters of DRL algorithms, where the DDPG algorithm was selected for investigation. The WOA was selected as an appropriate swarm-based optimization algorithm since WOA has a high significance when compared to other optimization approaches due to its exploitation, exploration, and ability to get rid of local minima [34]. The selected set of DDPG hyperparameters to be optimized are (actor learning rate – critic leering rate – batch size - discount factor – soft update parameter). Those hyperparameters must be predefined before training began. Results showed that optimized DDPG hyperparameters consistently returned higher total rewards at test time than commonly used reference hyperparameter suggested by an expert. The efficiency of DRL algorithms has been recognized to be sensitive to their hyperparameters. We witnessed multiple search agents’ training with different hyperparameter combinations regarding the optimization method, therefore the DDPG’s hyperparameters sensitivity was noticeable. This illustrates the challenge of optimizing the DDPG algorithm’s hyperparameters, as one search agent could be stuck in a poor policy for a long period if the agent initialized with an inappropriate hyperparameter set. Since DRL algorithms are sensitive to small modifications in their hyperparameters, precautions should be taken when optimizing them. For efficient optimization, we believe that splitting the search space (selecting the number of whales and the number of iterations) and determining the range of each hyperparameter must be accurately determined.

It was also obvious that randomization plays an important role in exploration and exploitation, which is at the heart of WOA; therefore, using the current randomization technique in WOA increases computational time, particularly for highly complex problems [65], which is the case in AD. Furthermore, the WOA algorithm’s convergence and speed are dependent on one control parameter, which is (a). This parameter has a significant impact on WOA’s efficiency [66]. As a result of the mentioned factors, we discovered that WOA has a slow convergence rate during both the exploration and exploitation phases [67].

Our findings show that the WOA can find hyperparameters that achieve the optimal strategy by maximizing the gained accumulated reward along with training episodes as we investigated the applicability of the proposed method within an AD environment represented in the TORCS simulation. In the future, we can perform experiments to analyze how hyperparameter optimization techniques act in model-based reinforcement learning algorithms, where DRL methods are effectively modeling the given environment. The model-based RL has been observed more productive than the model-free RL. By comparing model-based RL with model-free algorithms for task-specific performance, sometimes model-based RL yields lower results. In the future, we will investigate if the capabilities of model-based RL techniques would be enhanced by optimizing their hyperparameters.
